# Coordination of PRKCA/PRKCA-AS1 interplay facilitates DNA methyltransferase 1 recruitment on DNA methylation to affect protein kinase C alpha transcription in mitral valve of rheumatic heart disease

**DOI:** 10.1080/21655979.2021.1971482

**Published:** 2021-09-05

**Authors:** Zan Xie, Qianli Wang, Shaojuan Hu

**Affiliations:** a Department of Cardiology, The Affiliated Yantai Yuhuangding Hospital of Qingdao University, Yantai City, China; b Cardiovascular Surgery Intensive Care Unit, The Affiliated Yantai Yuhuangding Hospital of Qingdao University, Yantai City, China

**Keywords:** Rheumatic heart disease, protein kinase c alpha, dna methylation, prkca-as1, dnmt1

## Abstract

In the present study, mitral valve tissues from three mitral stenosis patients with RHD by valve replacement and two healthy donors were harvested and conducted DNA methylation signature on PRKCA by MeDIP-qPCR. The presence of hypomethylated CpG islands at promoter and 5ʹ terminal of PRKCA was observed in RHD accompanied with highly expressed PRKCA and down-regulated antisense long non-coding RNA (lncRNA) PRKCA-AS1 compared to health control. Furthermore, the enrichments of DNMT1/3A/3B on PRKCA were detected by ChIP-qPCR assay *in vivo* and in human cardiomyocyte AC16 and RL-14 cells exposed to TNF-α *in vitro*, and both demonstrated that DNMT1 substantially contributed to DNA methylation. Additionally, PRKCA-AS1 was further determined to bind with promoter of PRKCA via 5ʹ terminal and interact with DNMT1 via 3ʹ terminal. Taken together, our results illuminated a novel regulatory mechanism of DNA methylation on regulating PRKCA transcription through lncRNA PRKCA-AS1, and shed light on the molecular pathogenesis of RHD occurrence.

## Introduction

Rheumatic heart disease (RHD) is an autoimmune inflammatory disease responding to group A streptococcal infection, which results in mitral valve damage and hemodynamic changes. RHD remains a health burden in developing countries [1]. Although the progression of valve surgery has improved the clinical treatment for RHD, frequent serious complications and irreversible valve dysfunction still challenges the living quality of patients owing to the failure of early detection [2].

DNA methylation as the most extensively epigenetic study is regulated by DNA methyltransferases (DNMTs) family, and controls gene expression without DNA sequence changes. In general, DNA methylation negatively governs transcription activity via impacting with polarity of promoter coordinated with multiple transcription factors and epigenetic regulators [3]. The mode of action of different DNMTs for DNA methylation catalysis is distinct. DNMT1 is responsible for hemimethylated modification in cell mitosis whereas DNMT3A/B are essential for *de novo* methylation in zygotic and embryonic development [4]. Nevertheless, the ability of CpG sites recognition and the efficiency of DNA methylation process by DNMTs seem to be less studied.

Increasing evidences have reported that abnormal DNA methylation plays an important role in RHD development and progression [5,6]. Previous study has characterized the phenotype of global differentially methylated regions in RHD *in vivo* and identified protein kinase C alpha (PRKCA) as one of the candidate genes whose DNA methylation is associated with the pathogenesis of RHD [7]. However, the underlying mechanism of DNA methylation on PRKCA transcription behind the RHD occurrence remains unclear.

In this study, we take PRKCA for example, and mainly focuses on the regulatory roles of DNMTs in PRKCA in RHD. Our results may provide a common research model of studying a single gene on DNA methylation, and advance the understanding on the epigenetic regulation for RHD pathogenesis.

## Methods

### Specimen collection

Three RHD patients (two males and one female) who received mitral valve replacement in Yantai Yuhuangding Hospital from April 2016 to May 2018 were enrolled in this study as the following criteria: I) Diagnosis of mitral valve stenosis; II) left ventricular ejection fraction volume >50% and left ventricular end-diastolic diameter <55 mm; III) Antistreptolysin O > 500 U, positive C reactive protein; IV) no history of cardiomyopathy, congenital heart disease, liver disease or renal disease. Healthy mitral valve tissues were obtained from two remains donation by traffic accidents. The average age of participants is 66.85 ± 7.33 year. All the participants and their families understood and signed the informed consent. The documents of signed informed consent and Ethics Committee of Yantai Yuhuangding Hospital were all provided to approve this study. The freshly isolated human tissues were washed by saline and stored in −80°C for the subsequent experiments.

### Cell culture

Human cardiomyocyte-like AC16 cells [8] and fetal ventricular cardiomyocyte RL-14 cells [9] obtained from Type Culture Collection of the Chinese Academy of Sciences (Beijing, China) were cultured in F-12 K medium (Gibco, Carlsbad, CA, USA) supplemented with 12.5% fetal bovine serum (Gibco). The conditions of 100 ng/mL tumor necrosis factor-α (TNF-α) (APExBIO, Houston, TX, USA) for 16 h [10], 20 ng/mL 5-Azacytidine (APExBIO) for 24 h [11], 1 μM p38/MAPK inhibitor (2-(4-chlorophenyl)-4-(4-fluorophenyl)-5-(pyridin-4-yl)-1H-pyrazol-3(2 H)-one) (APExBIO) for 4 h, 50 mM transforming growth factor β1 (TGFB1) inhibitor (4-[2-(6-methylpyridin-2-yl)-5,6-dihydro-4 H-yrrolo[1,2-b]pyrazol-3-yl]quinoline-6-carboxamide) (APExBIO) for 4 h, 0.1 μM Rho associated coiled-coil containing protein kinase 1/2 (ROCK1/2) (4-[(1 R)-1-aminoethyl]-N-pyridin-4-ylcyclohexane-1-carboxamide) (APExBIO) for 6 h, 10 μM peroxisome proliferator activated receptor gamma/delta (PPARγ/δ) (2,5-dichloro-N-(2-methyl-4-nitrophenyl) benzenesulfonamide) (APExBIO) for 4 h, and 100 μM signal transducer and activator of transcription 3 (STAT3) (5,15-diphenyl-21 H,23 H-porphine) (APExBIO) for 6 h were adopted. Nucleotides for siRNAs of DNMT1, DNMT3A, DNMT3B as previously described [12]. PRKCA-AS1 RNA interference were synthesized from GenePharma (Shanghai, China). The full length and truncated PRKCA-AS1 was synthesised and cloned into pCMV-HA plasmid (631,604, Addgene, Watertown, MA, USA) by Sangon Biotech (Shanghai, China). 1x10^6^ cells were transfected with plasmids using Lipofectamine 3000 (Thermo Fisher Scientific, Waltham, MA, USA) for 24 h. The conditions of small molecular drugs or recombined proteins treatment were listed in Table S1.

### Enzyme-linked immunosorbent assay (ELISA)

The levels of biochemical indicators in the serum were determined, including interleukin (IL)-1β (E-CL-H0141, Elabscience, Houston, TX, USA) and IL-4 (E-EL-H0101, Elabscience) in serum according to the manufacturer’s instructions.

### Methylated DNA/chromatin/RNA immunoprecipitation (MeDIP/ChIP/RIP) assay

For MeDIP assay [13], genomic DNA from tissues or 5x10^6^ cells was extracted by phenol chloroform. For ChIP assay [14], tissues or 5x10^6^ cells were fixed with 1% formaldehyde, quenched with 0.125 M glycine at room temperature and then lysed in 500 μl of lysis buffer (10 mM Tris-HCl (pH 8.0), 10 mM NaCl, 0.2% NP40 and 100 U/ml protease inhibitor cocktail) on ice for 30 min, and collected supernatant after 14,000 rpm centrifugation. For RIP assay [15], tissues or 5x10^6^ cells were added 1 ml nuclear isolation buffer (1.28 M sucrose, 40 mM Tris-HCl (pH 7.5), 20 mM MgCl_2_, 4% Triton X-100) on ice for 20 min for nucleus isolation. After 2500 g centrifugation for 15 min, pellet was resuspended by 1 ml RIP buffer (150 mM KCl, 25 mM Tris-HCl (pH 7.4), 5 mM EDTA, 0.5 mM DTT, 0.5% NP40, 100 U/ml RNAase inhibitor and protease inhibitor cocktail).

DNA or RNA was mechanically sheared by Bioruptor Pico 60 KHz Sonicator (Diagenode, Seraing, Belgium). Ten percent of whole lysate was stored as input, whereas the rest of it was incubated with 1 μg primary antibodies of interest including 5-Methylcytosine (Cat. No. 61,479, Activemotif, Carlsbad, CA, USA), DNMT1 (NB100-56,519, Novus, Centennial, CO, USA) and SMAD2 (NBP2-67,376, Novus) at 4°C overnight. Additional 2-h pull down by protein A/G beads (Thermo Fisher Scientific) was performed at 4°C with gentle rotation. Beads were washed by the appropriated buffer for three times. Coprecipitated RNA was isolated by RNAiso (Takara, Tokyo, Japan). Coprecipitated DNA was purified by phenol chloroform methods. Primers designed to encompass approximately 150 bp around the target regions were used to detect the enrichment using qPCR.

### RNA isolation

RNA was extracted using RNAiso reagent, quantified with a NanoDrop (Thermo Fisher Scientific). 100 ng RNA was conversed into cDNA using a reverse transcription kit (Roche, Basel, Switzerland). PRKCA and PRKCA-AS1 expressions were detected by qPCR.

### Quantitative PCR (qPCR) assay

The DNA templates were detected using Fast Universal SYBR Green Real-time PCR Master Mix (Roche) under the following conditions: 95°C/2 min and 50 cycles of 95°C/5 s, 60°C/10 s, 72°C/ 30 s, and 72°C/10 min. GAPDH was used for the loading control. All primers used in this study are listed in Table S2.

### Western blot (WB) assay

5x10^6^ cells were added the 500 µl RIPA buffer (Thermo Fisher Scientific) with protease inhibitor cocktail (Beyotime Biotechnology, Shanghai, China), and quantified the concentration using BCA methods. Aliquots of proteins (40 µg) were added into the lanes of 10% SDS polyacrylamide gel, and the proteins were separated through electrophoresis and transferred onto nitrocellulose membranes. Subsequently, the membranes were congested with 5% nonfat dry milk in 0.01 M PBS buffer (pH 7.4) and 0.05% Tween-20 for 1 h at room temperature (RT). The blocked membranes were then incubated with primary antibodies of PRKCA (1:2000, NB600-201, Novus), DNMT1 (1:2000, the same antibody with ChIP), DNMT3A (1:1500, NB100-265, Novus), DNMT3B (1:2000, NB300-516, Novus) and GAPDH (1:5000, AF5009, Beyotime Biotechnology) overnight at 4°C, followed by incubation with the appropriate secondary antibodies (horseradish peroxidase-conjugated rabbit anti-mouse diluted with 1: 10,000 and donkey anti-rabbit diluted with 1: 5000, Beyotime) for 30 min at RT. The expression was determined by enhanced chemiluminescence method using Amersham Imager 600 system (GE Healthcare Life Sciences, Pittsburgh, PA, USA) whereas the density of the immunoblots was measured with Quantity One 4.62 software (Bio-Rad Laboratories, Hercules, CA, USA).

### Fluorescence in situ hybridization (FISH)

The FISH probes designed for PRKCA labeled with R-Phycoerythrin (excitation wavelength 565 nm) and for PRKCA-AS1 labeled with FITC (excitation wavelength 495 nm) were purchased from G&P Medical Enterprises (Beijing, China). Probes sequences can be found in Table S2. 40 μm mitral valve tissues sections or slides filled with AC16 cells washed by PBS were hypotension treatment in 0.075 M KCl at 37°C for 25 min, fixed by pure ethanol for 10 min and dropped on the glass slide, then aged at 56°C for 60 min. The slides were washed by PBS twice and dehydrated with the 70, 85, and 100% ethanol for 3 min in order, and naturally dried. 10 μl probes were added on and mounted immediately. Slides were next denatured 75°C for 5 min and 42°C for 16 h, then removed the cover glass and incubated in 2 × SSC for 5 min and 0.1% NP-40/2 × SSC for another 2 min at 46°C, followed by 70% ethanol at RT for 3 min and dried in dark. 15 μl DAPI were dropped and mounted again. After 10 min incubation in dark, the slides were observed under the fluorescence microscope with the appropriate filters.

### Statistical analysis

All statistical analyses were conducted in SPSS 20 (IBM, Armonk, NY, USA). For qPCR data, the 2^delta delta method was used to calculate the expression and protein enrichment on DNA. Student’s t-test was used to evaluate differences between groups. A *p*-value less than 0.05 was considered to indicate a significant difference.

## Results

To investigate the epigenetic role of PRKCA in RHD, we characterized the DNA methylation pattern of PRKCA in RHD *in vivo* and *in vitro*, and further found that the abnormal DNA methylation was ascribed to DNMT1 binding affinity. Antisense lncRNA PRKCA-AS1 was determined to connect and recruit DNMT1 on the promoter of PRKCA. Finally, we further investigated the effect of p38/MAPK pathway on PRKCA-AS1 expression in RHD.

### Characterization of DNA methylation on PRKCA in mitral valve of RHD

**Figure 1.**
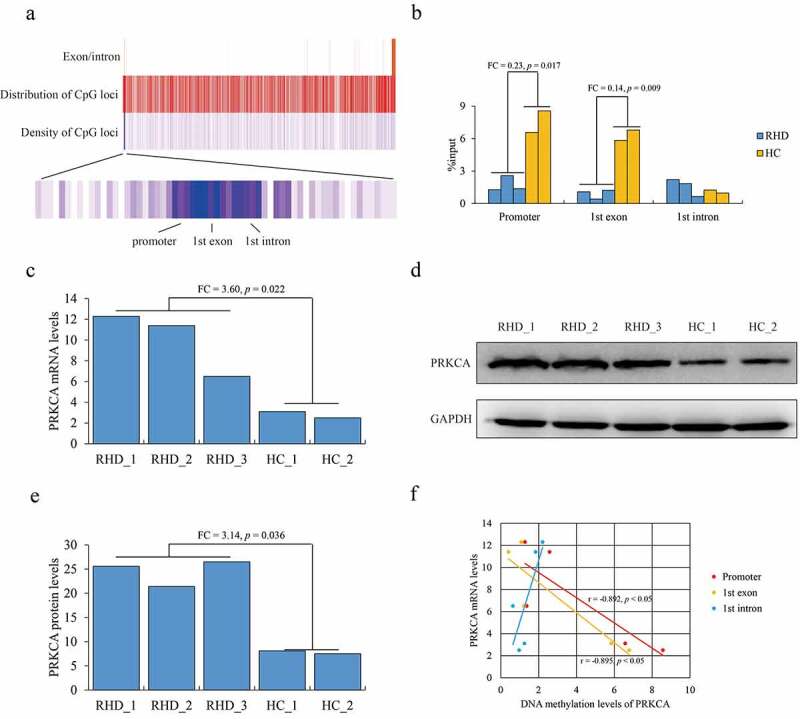
DNA methylation of PRKCA in mitral valve of RHD. (a) the overview of CpG loci distribution within human PRKCA gene. (b) DNA methylation on 5ʹ terminal of PRKCA (promoter, first exon and first intron) in mitral valve of RHD by MeDIP-qPCR assay. (c) PRKCA transcription levels in mitral valve of RHD by qPCR assay. (d) PRKCA protein levels in mitral valve of RHD by WB assay. (e) PRKCA protein levels calculated by gray intensity analysis. (f) the correlation between DNA methylation and PRKCA transcription by pearson correlation analysis. the given data was processed as mean ± standard error and compared between RHD and HC groups by student’s t-test. ‘RHD’: rheumatic heart disease; ‘HC’: healthy control; ‘FC’: fold change

First, we investigated the DNA methylation signature of PRKCA in mitral valve tissues of RHD by MeDIP-qPCR. We observed that ‘CG’ loci with highest density were distributed at −590 to +648 containing promoter, first exon and partial first intron of PRKCA (consider ‘A’ of ‘ATG’ as +1) (Figure 1(a)), which were majorly concentrated on to investigate DNA methylation. The overall DNA methylation levels at these detected regions of PRKCA showed that PRKCA was hypomethylaed in RHD compared to health control (Fold change (FC) = 0.28, *p* = 0.021), where DNA methylation of promoter (FC = 0.23, *p* = 0.017) and the first exon (FC = 0.14, *p* = 0.009) of PRKCA was significantly lower in RHD compared to health control (Figure 1(b)). The mRNA (FC = 3.60, *p* = 0.022) (Figure 1(c)) and protein levels (FC = 3.14, *p* = 0.036) (Figure 1(d-e)) of PRKCA were both highly expressed in RHD compared to healthy control. Consistently, Pearson correlation analysis showed that DNA methylation at promoter (r = −0.892, *p* < 0.05) and exon (r = −0.895, *p* < 0.05) were strongly negatively correlated with PRKCA transcription (figure 1(f)). Taken together, we determined that the abnormally hypomethylated PRKCA especial at 5ʹ terminal was closely related to up-regulation of PRKCA in mitral valve tissues of RHD.

### DNMT1 contributes to DNA methylation on promoter of PRKCA

**Figure 2.**
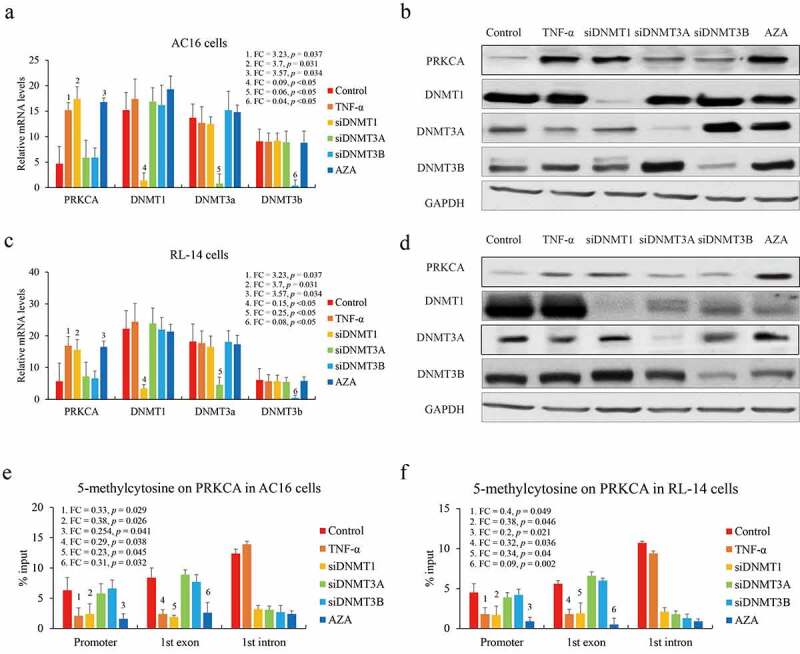
The effect of DNMTs on DNA methylation of PRKCA in human cardiomyocyte cells. (a) the mRNA and (b) protein of PRKCA in AC16 cells induced by TNF-α, DNMTs RNA interference and 5-azacytidine. (c) the mRNA and (d) protein of PRKCA in RL-14 cells induced by TNF-α, DNMTs RNA interference and 5-azacytidine. DNA methylation on 5ʹ terminal of PRKCA (promoter, first exon and first intron) in (e) AC16 and (f) RL-14 cells induced by TNF-α, DNMTs RNA interference and 5-azacytidine. the given data from triplicate experiments was processed as mean ± standard error and compared by student’s t-test. No. 1 to 6 indicate six comparison with control group. ‘FC’: fold change; ‘si’: siRNA; ‘AZA’: 5-azacytidine

Although DNMTs family contribute to DNA methylation, different catalytic means of DNMT1 (maintenance methylation) and DNMT3A/B (*de novo* methylation) have been declared. Next, we investigated which DNMTs were responsible for regulating gene body methylation of PRKCA. Two human cardiomyocyte cell lines AC16 and RL-14 were exposed to 100 ng/mL TNF-α for mimicking the *in vitro* inflammatory model or direct DNMTs knockdown via RNA interference or 20 ng/mL 5-Azacytidine (DNMTs inhibitor) treatment as a positive control. IL-1β and IL-4 levels in medium were detected by ELISA to confirm the inflammatory effect of TNF-α in cardiomyocyte cells (Figure S1). We observed that PRKCA was up-regulated after TNF-α treatment (AC16: FC = 3.23, *p* = 0.037; RL-14: FC = 2.96, *p* = 0.04) or DNMT1 deficiency (AC16: FC = 3.7, *p* = 0.031; RL-14: FC = 2.74, *p* = 0.047) or 5-Azacytidine addition (AC16: FC = 3.57, *p* = 0.034; RL-14: FC = 2.89, *p* = 0.042) both in AC16 (Figure 2(a-b)) and RL-14 cells (Figure 2(c-d)). Moreover, we also observed that DNA methylation levels on promoter and first exon of PRKCA substantially decreased after TNF-α (Promoter: AC16: FC = 0.33, *p* = 0.029; RL-14: FC = 0.4, *p* = 0.049; first exon: AC16: FC = 0.29, *p* = 0.038; RL-14: FC = 0.32, *p* = 0.036) or DNMT1 deficiency (Promoter: AC16: FC = 0.38, *p* = 0.026; RL-14: FC = 0.38, *p* = 0.046; first exon: AC16: FC = 0.23, *p* = 0.045; RL-14: FC = 0.34, *p* = 0.04) or 5-Azacytidine treatment (Promoter: AC16: FC = 0.29, *p* = 0.038; RL-14: FC = 0.32, *p* = 0.036; first exon: AC16: FC = 0.31, *p* = 0.032; RL-14: FC = 0.09, *p* = 0.002) compared to negative control and DNMT3A/B knockdown in AC16 (Figure 2(e)) and RL-14 cells (figure 2(f)). Unexpectedly, protein levels of all DNMTs had almost unchanged induced by TNF-α compared to negative control either in AC16 or RL-14 cells (Figure 2(b-d)), which suggested that DNMT1 was likely a partial but not an essential factor for PRKCA induction by TNF-α. Results above indicated that DNMT1 contributed to DNA methylation on 5ʹ terminal negatively correlating with PRKCA transcription in RHD.

### The interaction between 5ʹ terminal of PRKCA-AS1 and PRKCA

**Figure 3.**
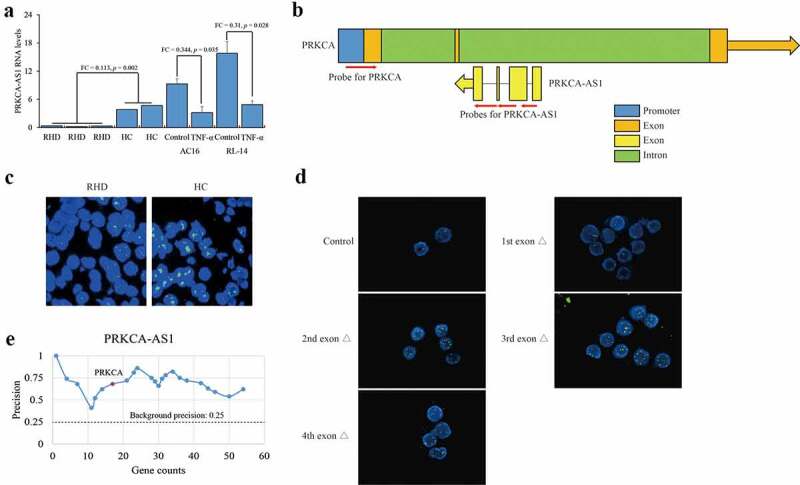
Interaction between PRKCA-AS1 and PRKCA in RHD. (a) the RNA levels of PRKCA-AS1 in mitral valve of RHD tissues, TNF-α-induced AC16 and RL-14 cells. the given data was processed as mean ± standard error and compared between RHD and HC groups by student’s t-test. (b) the overview of PRKCA and PRKCA-AS1 location. blue: promoter; orange: exon of PRKCA; green: intron of PRKCA; yellow: exon of PRKCA-AS1; red arrows: the probes for FISH assay; orange and yellow arrows: the transcription orientation. (c) the staining for PRKCA-AS1 transcripts and PRKCA genomic region in RHD with 400 x magnification by FISH assay. (d) the staining for PRKCA-AS1 transcripts and PRKCA genomic region in AC16 cells with different truncations of exogenous PRKCA-AS1 with 400 x magnification by FISH assay. red: the staining for allele PRKCA in genomic DNA; green: the staining for PRKCA-AS1 transcripts; yellow: the overlap of two kinds of probes. (e) the computational examination of PRKCA-AS1 for target promoter of genes by ‘triplex domain finder’ tool. ‘FC’: fold change; ‘Δ’: deletion

In general, DNMTs recognize unmethylated CpG loci and transfer methyl group for DNA methylation catalysis. Although DNMTs expression did not change, DNA methylation on PRKCA was weakened after TNF-α stimulation, suggesting an unknown epigenetic regulation coordinated with DNMT1 for DNA methylation on PRKCA. Previous studies have indicated that the antisense lncRNAs can link with DNA and transcription factors, and govern gene expression via chromatin organization [12,16,17], which enlighten us on the current system. To figure out the underlying mechanism of DNMT1 on PRKCA transcription, we focused on the antisense lncRNA PRKCA-AS1, and investigated their expression in RHD. PRKCA-AS1 was majorly expressed in nucleus and significantly down-regulated in RHD (FC = 0.113, *p* = 0.002) as well as in TNF-α treated AC16 (FC = 0.344, *p* = 0.035) and RL-14 cells (FC = 0.31, *p* = 0.028) compared to control (Figure 3(a)). Certainly, PRKCA-AS1 transcription was determined to be positively correlated with DNA methylation at promoter (RHD: r = 0.98, *p* = 0.003; AC16 cells: r = 0.412, *p* = 0.027; RL-14 cells: r = 0.541, *p* = 0.018) and first exon (RHD: r = 0.997, *p* < 0.05; AC16 cells: r = 0.366, *p* = 0.39; RL-14 cells: r = 0.324, *p* = 0.44) of PRKCA *in vivo* and *in vitro*. As an antisense lncRNA transcribed from the second intron of PRKCA, the potential interaction between PRKCA-AS1 and genomic region of PRKCA was investigated by FISH. Multiple single-strand DNA probes spanning across PRKCA promoter and PRKCA-AS1 introns were designed for PRKCA-AS1 and PRKCA (Figure 3(b)). Although the substantial overlapping between PRKCA-AS1 and PRKCA was observed both in RHD and healthy control *in vivo* (Figure 3(c)), it is still hard to figure out the precise position of PRKCA-AS1 for PRKCA interaction. To this end, PRKCA-AS1 with respective truncation of four exons were transfected into AC16 cells, and the disappearance of affinity by the first exon absence compared to control and the other three truncated fragments of PRKCA-AS1 (Figure 3(d)) indicated that 5ʹ terminal of PRKCA-AS1 indeed bound with PRKCA. Interestingly, given the computational examination of PRKCA-AS1 by ‘Triplex Domain Finder’ tool (http://www.regulatory-genomics.org/tdf/basic-introduction/) [18], promoter of PRKCA was also included in the putative genomic targets of PRKCA-AS1 (Figure 3(e)). The results above suggested that PRKCA-AS1 was proposed to bind with PRKCA.

### DNMT1 recruitment to PRKCA promoter facilitated by 3ʹ terminal of PRKCA-AS1

**Figure 4.**
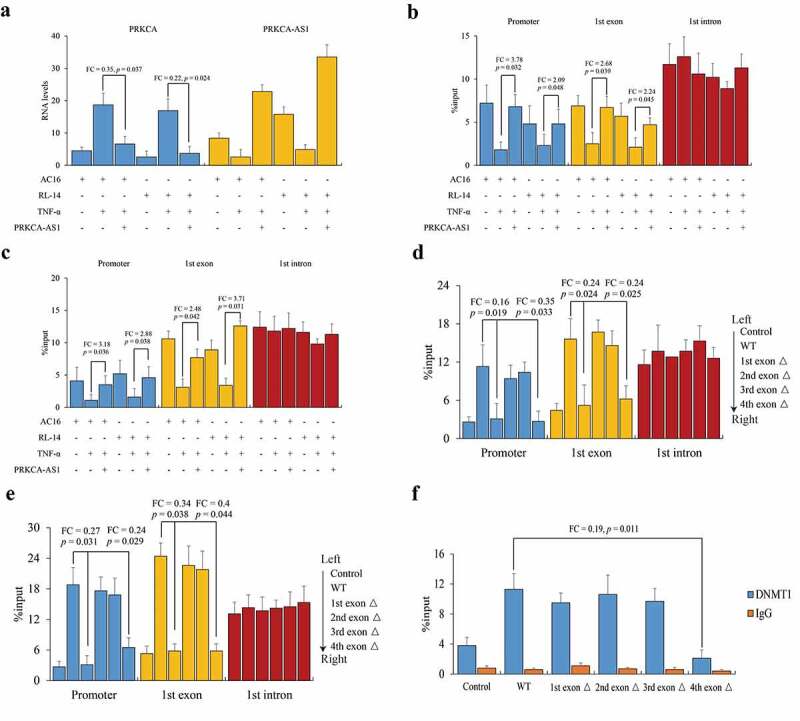
The role of PRKCA-AS1 in regulating PRKCA expression in RHD. (a) the RNA levels of PRKCA-AS1 in TNF-α-induced AC16 and RL-14 cells. (b) DNA methylation and (c) DNMT1 occupancy on 5ʹ terminal of PRKCA (promoter, first exon and first intron) in TNF-α-induced AC16 and RL-14 cells. (d) DNMT1 occupancy and (e) DNA methylation on 5ʹ terminal of PRKCA (promoter, first exon and first intron) in TNF-α-induced AC16 and RL-14 cells treated with different truncations of exogenous PRKCA-AS1. (f) interaction between DNMT1 and different fragments of PRKCA-AS1 by RIP-qPCR assay. the given data from triplicate experiments was processed as mean ± standard error and compared by student’s t-test. ‘FC’: fold change; ‘Δ’: deletion

Since PRKCA-AS1 was declined after TNF-α treatment, now we started to investigate the role of PRKCA-AS1 in regulating PRKCA transcription. We in turn further over-expressed PRKCA-AS1, and determined that PRKCA mRNA was remarkably down-regulated in AC16 (FC = 0.35, *p* = 0.037) and RL-14 cells (FC = 0.22, *p* = 0.024) compared to TNF-α treatment (Figure 4(a)), indicating an effect of PRKCA-AS1 against the inflammatory induction. Then, we conducted MeDIP-qPCR to investigate DNA methylation changes by PRKCA-AS1 induction. With the exception of intron, DNA methylation levels on promoter (AC16: FC = 3.78, *p* = 0.032; RL-14: FC = 2.09, *p* = 0.048) and first exon (AC16: FC = 2.68, *p* = 0.039; RL-14: FC = 2.24, *p* = 0.045) of PRKCA were both elevated concurrent with higher DNMT1 occupancy (AC16: promoter: FC = 3.18, *p* = 0.036; exon: FC = 2.48, *p* = 0.042; RL-14: promoter: FC = 2.88, *p* = 0.038; exon: FC = 3.71, *p* = 0.031) by PRKCA-AS1 over-expression (Figure 4(b-c)). Likewise, truncated PRKCA-AS1 was individually transfected into AC16 cells, and we observed that the exogenous PRKCA-AS1 source could substantially improve DNMT1 and DNA methylation of PRKCA. It is noteworthy that PRKCA-AS1 with the first (promoter: FC = 0.16, *p* = 0.019; first exon of PRKCA: FC = 0.24, *p* = 0.024) and fourth (promoter: FC = 0.35, *p* = 0.033; first exon of PRKCA: FC = 0.24, *p* = 0.025) exons deficiency resulted in the loss of DNMT1 recruitment (Figure 4(d)). Similarly, the consequent DNA methylation was compromise by first (promoter: FC = 0.27, *p* = 0.031; first exon of PRKCA: FC = 0.34, *p* = 0.038) and fourth (promoter: FC = 0.24, *p* = 0.029; first exon of PRKCA: FC = 0.40, *p* = 0.044) exons deficiency of PRKCA-AS1 compared to wild type (Figure 4(e)). Interestingly, RIP-qPCR assay of DNMT1 indicated that only PRKCA-AS1 with fourth exon deficiency failed to connect with DNMT1 compared to wild type (FC = 0.19, *p* = 0.011) and the other truncations (figure 4(f)), indicating that 3ʹ terminal of PRKCA-AS1 contributed to DNMT1 interaction.

### Activated p38/MAPK pathway suppresses PRKCA-AS1 expression in RHD

**Figure 5.**
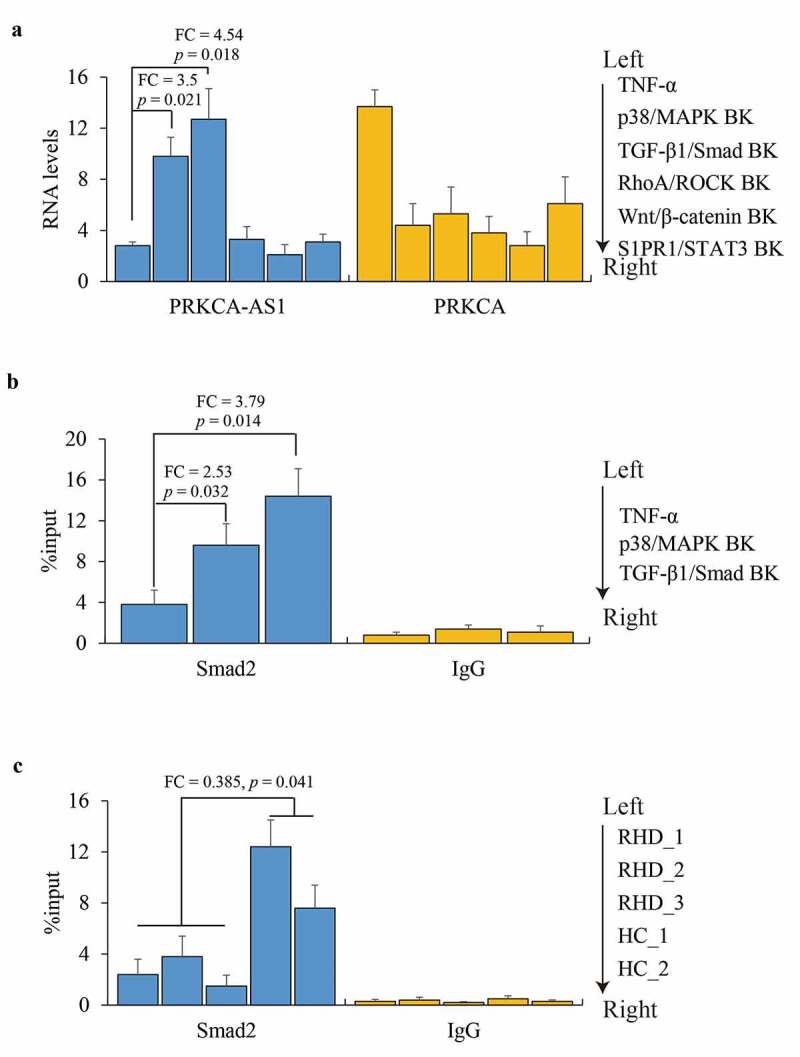
The role of p38/MAPK in regulating PRKCA-AS1 expression in RHD. (a) the RNA levels of PRKCA-AS1 and PRKCA in TNF-α-induced AC16 cells treated with inhibition of different signaling pathways. (b) Smad2 occupancy on PRKCA-AS1 in TNF-α-induced AC16 cells treated with inhibition of different signaling pathways. (c) Smad2 occupancy on PRKCA-AS1 in mitral valve of RHD. the given data from triplicate experiments was processed as mean ± standard error and compared by student’s t-test. ‘FC’: fold change; ‘BK’: blocking

Finally, the regulatory mechanism on PRKCA-AS1 was explored. Based on previous review that RHD was implicated in activation of four intracellular p38/MAPK/TGF-β1/Smad, RhoA/ROCK/Akt, Wnt/β-catenin and S1PR1/STAT3 signaling pathways [19], AC16 cells were treated with TNF-α, followed by five kinds of protein inhibitors or agonists for p38/MAPK, TGFB1, ROCK1/2, PPARγ/δ and STAT3 respectively. We observed that only p38/MAPK (FC = 3.5, *p* = 0.021) and TGFB1 (FC = 4.54, *p* = 0.018) blocking could enhance PRKCA-AS1 transcription although all these pathways inhibition caused PRKCA reduction against the effect of TNF-α (Figure 5(a)). Moreover, ChIP-qPCR assay unmasked that a robust occupancy of Smad2 was observed on the upstream of PRKCA-AS1 at the second intron of PRKCA in suppression of p38 (FC = 2.53, *p* = 0.032) and TGFB1 (FC = 3.79, *p* = 0.014) compared to TNF-α (Figure 5(b)). Consistently, the enrichment of Smad2 validated *in vivo* was weakened in RHD compared to healthy control (FC = 0.385, *p* = 0.041) (Figure 5(c)). The outcomes above indicated that TNF-α might enhance PRKCA-AS1 transcription through p38/MAPK/TGF-β1/Smad pathway.

## Discussion

The pathogenesis of RHD is the result of valve damage caused by an abnormal immune response consisting of humoral and cellular components after exposure to group A streptococcal infection [1]. The role of autoimmune reactions in the pathogenesis of acute rheumatic fever was demonstrated when antibodies against group A streptococcus cross-recognize to a series of cardiac proteins such as cardiac myosin, tropomyosin, keratin, laminin and vimentin, and multiple cytokines including IL-1, IL-6, and TNF-α exerted inflammatory effects on cardiomyocytes. In our study, TNF-α is used to prepare an *in vitro* environment of inflammatory reaction. We observe that both NF-κB and JAK/STAT signaling pathways are all activated, which indicates that the ectopic cytokine stimulators can mimic rheumatism to induce auto inflammation in myocardial cells.

PRKCA has been associated with emotional memory formation, posttraumatic stress syndrome, cancer, and aging. PRKCA as a key regulator of cardiac contractility is reported to be implicated in heart failure risks and treatment outcomes [20]. The suppression of PRKCA may fit the criteria of a therapeutic target with milder systemic effects that still boosts contractility in HF patients. This increase in PKCα activity is perplexing because it is also accompanied by up-regulation of a molecular braking mechanism. Previous studies revealed that differentially methylated PRKCA was both involved in development of fibromyalgia and RHD, which were usually caused by inflammatory cytokines and suffered from rheumatic symptoms [7,21]. Consistently, our data determines a hypomethylation pattern of PRKCA in RHD *in vivo* and *in vitro*. Moreover, we verify that transcription of PRKCA is up-regulated after DNMT1 knockdown, which suggests that the promoter and 5ʹ terminal methylated by DNMT1 contributes to regulating PRKCA transcription, and the other CpG loci on PRKCA showing no significant association with PRKCA transcription may not affected RNA polymerase. In RHD, previous studies reported a controversial phenotype of DNMTs expression [22,23]. In this case, we announce that each DNMT manages DNA methylation pattern in different CpG loci, which suggests that the target strategy for the certain DNMT is superior to the classical DNMT inhibitors, which usually affect the global DNA methylation and cause more side reaction.

Antisense lncRNA regulates the adjacent target gene via RNA-DNA interaction has been determined previously. X-chromosome inactivation by lncRNA Xist is one of the most classic case [24,25]. Besides, lncRNA can interact with transcription factors or enzymes in nucleus to modulate gene expression has also been reported [26–28]. Our results indicate that PRKCA-AS1 bridges PRKCA and DNMT1 through 5ʹ and 3ʹ terminals, respectively, and give one possible explanation for why DNMT1 can be recruited to promoter of PRKCA-AS1.

Although the sequences of PRKCA and PRKCA-AS1 are aligned but fail to find the identical sequence, the substantial interaction is confirmed by FISH assay, which implies that PRKCA-AS1 may recognize a certain DNA motif of PRKCA promoter through a complicate loop structure, and need to be clarified in future study. Moreover, FISH data shows the presence of PRKCA-AS1 transcript in nucleus and indicates that PRKCA-AS1 may bind multiple genomic regions via the similar DNA motif with PRKCA, which can be verified by high-throughput sequencing followed by pull down assay.

Finally, p38/MAPK/TGF-β1/Smad signaling pathway is determined to be the reason behind aberrant DNA methylation of PRKCA. The activated p38/MAPK/TGF-β1/Smad is able to suppress PRKCA-AS1, thereby retard DNMT1 recruitment to PRKCA promoter, and modulate DNA methylation and transcriptional repression similarly with a previous regulation model [29]. Interestingly, another study indicates an opposite effect that NF-κB/p65/MUC1-C complex occupy the promoters of DNMT1 and DNMT3B to drive their transcription, but not DNMT3A in carcinoma cells [30]. Taken together, the regulatory network bridging inflammatory signaling pathway and DNMTs seems to be complicate in different diseases.

## Conclusion

Overall, our data suggests that PRKCA can be hypermethylated by PRKCA-AS1-meidated DNMT1 through p38/MAPK/TGF-β1/Smad signaling pathway against RHD, which advance the understanding of pathogenesis and progression of RHD, and provide the potential therapeutic targets for RHD.

## Supplementary Material

Supplemental Material

## Data Availability

The datasets used and/or analyzed during the current study are available from the corresponding author upon reasonable request.

## RESEARCH HIGHLIGHTS

Regulatory mechanism of PRKCA in RHD

DNA methylation by lncRNA

Role of antisense lncRNA
