# Supplementary figures and images for: Coordination of PRKCA/PRKCA-AS1 interplay facilitates DNA methyltransferase 1 recruitment on DNA methylation to affect protein kinase C alpha transcription in mitral valve of rheumatic heart disease
Source: Bioengineered. 2021 Sep 5;12(1):5904–15. doi: 10.1080/21655979.2021.1971482 (PMC8806685; doi:10.1080/21655979.2021.1971482)

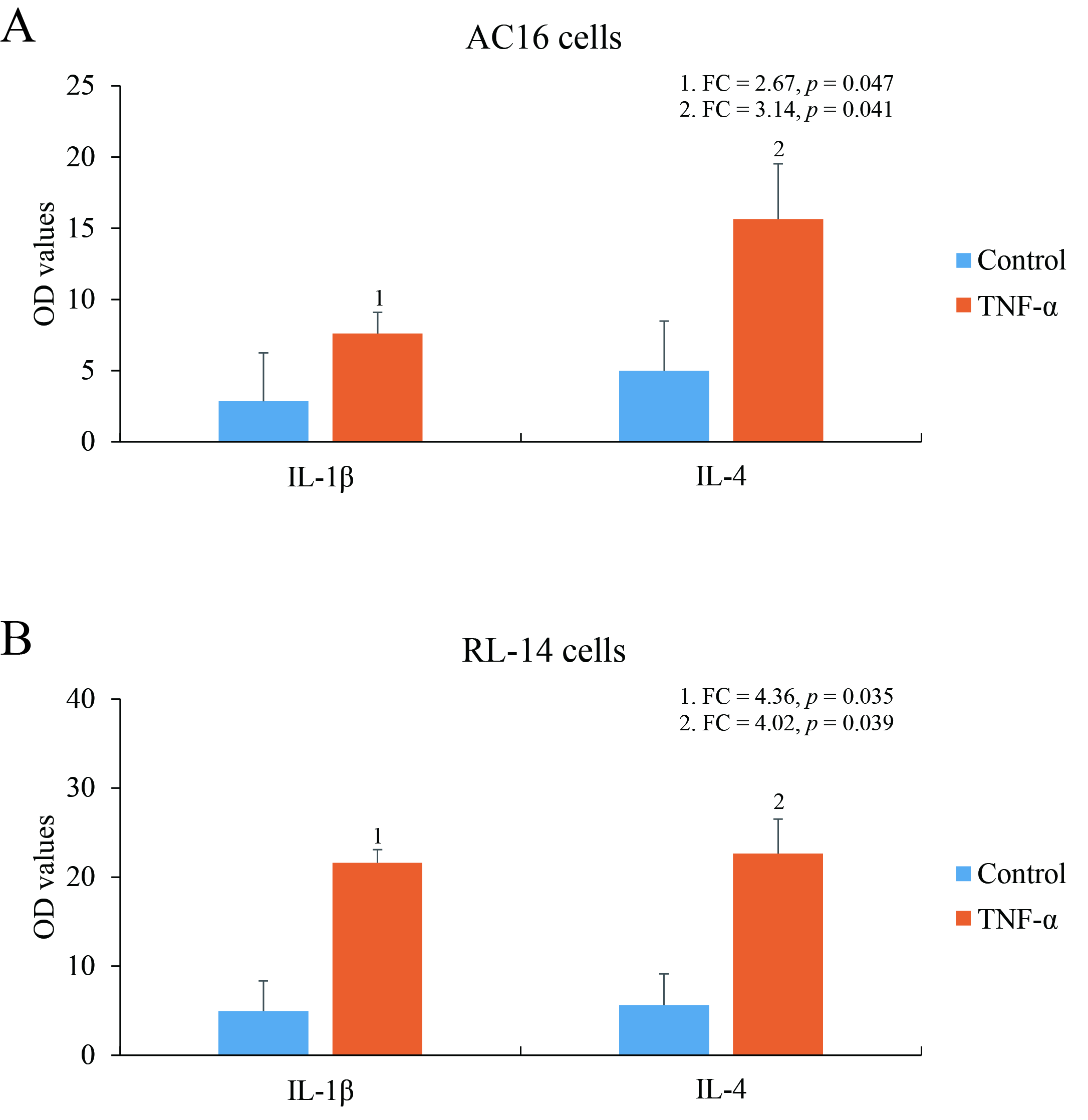

Supplement: Supplemental Material [file KBIE_A_1971482_SM8430.tif]
